# How Network Topologies Impact Project Alliance Performance: Evidence from the Movie Industry

**DOI:** 10.3390/e21090859

**Published:** 2019-09-03

**Authors:** Xin-Jie Zhang, Yong Tang, Jason Xiong, Wei-Jia Wang, Yi-Cheng Zhang

**Affiliations:** 1 School of Management and Economics, University of Electronic Science and Technology of China, Chengdu 610054, China; 2 School of Computer Science and Engineering, University of Electronic Science and Technology of China, Chengdu 610054, China; 3 Department of Physics, University of Fribourg, 1700 Fribourg, Switzerland; 4 Walker College of Business, Appalachian State University, Boone, NC 28608-2037, USA; 5 School of Information and Software Engineering, University of Electronic Science and Technology of China, Chengdu 610054, China

**Keywords:** social network analysis, sociophysics, movie project, box office, alliance network, centrality, structural holes

## Abstract

In many industries, partners are interconnected in project alliances that have limited lifespans and clearly-defined boundaries. The transparency of the movie industry provides a unique opportunity to study how alliance network topologies impact the performance of project alliances from the perspectives of social networks and organization theories. In this work, we compiled a massive movie dataset and constructed alliance networks for both movie production and distribution companies. Using the box office as the proxy for the financial performance of a movie project alliance, this research investigates how the two alliance networks impact the box office. We introduce the social network properties of degrees, centralities, and structural holes as alliance network variables into empirical regression models. The results show that alliance networks have a significant influence on the box office. The degrees of production companies and the structural holes of distribution companies are especially important to achieve success in the box office. The results add new evidence for the study of the movie economy and alliance networks. Meanwhile, this work also provides implications for the movie industry by revealing that it is essential to wisely choose partners that are appropriately embedded in alliance networks for the success of a movie project.

## 1. Introduction

Alliances formed by strategically-collaborating partners are common in a wide range of industries. These interorganizational systems have attracted studies from the perspective of economics, organizational science, and strategy management [1,2]. The previous literature investigated various types of alliance relationships like technology innovation, production development, supply chains, and joint ventures. By forming an alliance, partners can pool resources, exchange knowledge, share risks, enhance innovation capabilities, and expand markets [1]. For a focal firm, alliances established with multiple partners become an alliance portfolio for specific strategic objectives [3].

Most of these alliances are long-term, continuous, multipurpose, and strategical. However, some other alliances are formed for a specific purpose or a result orientated toward a limited lifespan. For instance, a construction project normally involves several partner companies to finish [4]. Large-scale software projects are usually developed by multiple developers and integrators [5]. These temporal and single-purpose cooperations are generalized as project alliances [6]. Typically, once a project goes through all stages, when final commercial values are realized, project alliances cease to exist and are disbanded to form new alliances for new projects [6]. Though project alliances exist in many domains, there is still a lack of studies on project alliances compared to the majority of the literature focusing on long-term alliances. In this project alliance study, the relationships of project performance, partner experience, and project entrepreneurship are investigated [6,7]. From the perspective of social network theory, partners form a network through cooperation activities involved in project alliances. Each time, new edges from a set of partners are added into the network when they establish a project alliance. For a given alliance, partners are connected and occupying certain positions in the network, laying the foundations for the appliance. The topological positions occupied by the partners on the network and the implicit social capital and structural holes obtained by them through the network are expected to affect the performance of the project alliance in a similar way to the common alliance networks [5,7,8,9,10,11].

There is much research on alliances in different industries, including construction [4], software [5], research and development [7], biotechnology [9,12], healthcare [10], and enterprises [11]. For the creative industry, movies and music recordings are the results of joint efforts of multiple partners in contract-based cooperations. Movies are especially typical projects involving the teamwork of a large number of individuals and companies working closely in roughly the same standard procedure [13]. Thanks to the public availability of detailed information about the alliances in the movie project lifetime, it provides a unique opportunity for researchers to look into the mechanisms of project alliances [14]. In the movie industry, production companies form a production alliance to co-finance and organize scripting, casting, filming, and post-production through the whole production procedure of a movie project. Once production is completed, multiple distribution companies form a distribution alliance to distribute the movie through theaters and other channels. Due to the limited lifespan of a movie project, the production alliance and the distribution alliance only exist as a temporary organization. However, a production alliance network between all production companies is formed through constantly emerging movie-centered production project alliances. A similar case applies for distribution alliances.

Undoubtedly, art achievements and word of mouth are essential for a movie as a performance art. However, as an industrial product of an alliance, the financial success or failure of a movie project concerns all participants. In line with the previous movie economy literature [15,16,17,18,19,20,21,22,23], we focused on the financial performance of a movie using the box office as the proxy of the performance of the movie project alliance. Even though the total revenue for a movie includes the box office and sales generated through a variety of channels like TVs, DVDs, and online websites, revenues from these sources tend to be highly correlated with the box office [24]. Moreover, publicly available box office data allow large-scale empirical studies. These suggest that focusing on the box office is sufficient and feasible. Movie projects are the joint efforts of production companies and distribution companies interconnected as alliance networks. The topological properties of alliance networks have a major influence on the performance of alliances. For a given movie, its production and distribution companies are located in production alliance networks and distribution alliance networks, respectively. Understanding how the embeddedness of companies influences financial performance is necessary for movie professionals.

The movie business has grown into a tremendous global industry with huge economic impact [13]. Recent years have witnessed a fast increase in budgets, intensified competitions, and unpredictable market uncertainties. All these factors turn the making of a movie into a highly risky project. The production alliance and distribution alliance pool resources by sharing risks, co-financing, assembling talents, and accessing distribution channels. It is essential to understand how these common practices in the form of alliances impact the performance of a movie project measured by the box office. There is sufficient literature on the determinants of movie success, such as sequel, genre, director, stars, screenplays, culture, and cast [16,18,25]. These characteristics are the most studied and widely considered as variables in studies. By introducing social network theory into the study of the economics of movies, the individual-level social networks of the cast are studied in the aspects of team composition, producer centrality, producer structural holes, old ties, and coreness [6,26]. In contrast, organizational-level alliance networks formed by production and distribution companies are less explored.

We used a set of collected movie industry data of 903 movies with the information of basic movie profiles, box office revenues, as well as their production companies and distribution companies. Alliance networks were constructed for production and distribution, respectively. The topological properties of the production alliance and distribution alliance, including degrees, eigenvector centralities, betweenness centralities, and structural holes, were investigated by establishing an empirical regression model.

The results showed that topological properties have different contribution levels to the box office. The degrees of production companies and the structural holes of distribution companies were found to contribute significantly and positively to the box office, while other properties were found to either negatively or not significantly contribute to the box office. This work contributes to the literature of the movie economy, project alliance networks, and alliance performance evaluations with implications for movie industry professionals. Moreover, the present work provides a new perspective to study the performance of project alliances by including the network properties of participants involved in project alliances as alliance network variables in regression models.

The rest of this paper is organized as follows. In Section 2, the movie project alliance is described with a focus on production alliance and distribution alliance. Section 3 develops the formal hypotheses. In Section 4, the data are described and summarized. Section 5 discusses the variables and empirical models. Section 6 reports the model results with a discussion. Finally, Section 7 summarizes the findings, contributions, practical implications, and the outlook of future research.

## 2. Movie Project Alliance

The modern movie industry has developed into a considerable global economy, creating millions of jobs and generating tremendous revenues annually. The uniformity of movie making and the openness of financial information provide unique opportunities for researchers of organization science, finance, economics, and strategy management to understand the movie industry [13]. A movie is a typical project-based business involving multiple stages and different stakeholders. As a joint project, the success of a movie requires the collaboration of a large number of individual talents, including directors, screenwriters, and stars, as well as a group of companies to organize and execute the movie project. Movie projects are becoming more complicated, consuming huge financial investments and time; all predictable and unpredictable factors contribute to the high risks and uncertainties. One thread of the literature focuses on the performance of a movie project, which is normally proxied by box office revenue [27,28,29,30,31]. In this line of research, the factors of the fundamental movie profile are considered, such as sequels [18,25], stars [17,32,33,34], genres [35], critics [15,19], awards [20,36], culture [20,37], seasonality [38], and word of mouth [16,22,39,40]. These empirical analyses reveal that fundamental profile factors have various degrees of impact on the success and performance of a movie. These studies also provide rich implications on marketing, cultural economics, consumer experience, and creative product design [13,41].

With the emergence of social network science [42,43], in recent years, we have witnessed a thread of studies on individual-level networks in the movie industry. Actors, directors, screenwriters, and other talents are modeled as social networks through cast and crew collaborations. The actors’ networks and their network properties were investigated [44,45]. However, their impact on movie profitability is less explored, even though networks carry important information to predict movie success [28].

The making of a movie not only relies on creative talents, but also depends on the contractual organizations of companies. Extended on profile factors, there is a thread of the literature that explored the factors of movie project alliances at the corporation level. The success of a movie is a long journey in which production and distribution are the primary two stages requiring substantial investment, complex organizations, and institutional arrangements [41]. In the production stage, investments and personnel are organized to finish movie making under the management of one or several production companies. These production companies form a movie project alliance to share resources, financial investments, and risks. In this production stage, the issues of risk reduction, co-financing, and market competition are investigated for production alliances [14]. Co-financing is to make movies with a large budget, and a production alliance can help to optimize release dates [14,46]. When a movie is finished, it faces challenges in the distribution stage, such as releasing, marketing, seasonality, and exhibitor channels [13,47,48]. This distribution process is highly costly, and carefully-organized resources are required to ensure maximized success in terms of box office revenues and social influence. All theater box offices are expected to be generated in a concise show-time window in competition with their competitors. Choosing capable distribution companies is key in this stage. Sometimes, the production company is also the distribution company of the same movie. However, it is common that distribution companies are formed as a distribution alliance for a movie to achieve box office success. These distribution alliances can strengthen bargaining powers over exhibitor channels to secure better timing and screens. This thread of studies contributes to the understandings of organizational learning and impermanence [49], the dynamics of creative organizations [50], and organizational forms [51].

In the movie industry, all movies and all production companies naturally form a bipartite graph. On the one side, any given movie is produced by one or multiple production companies. On the other side, a production company has one or more movies it has produced. If we collect and combine all historical production information, then we can obtain a complex network of all movies and production companies. For production companies, any two companies are indirectly connected by one or multiple coproduced movie projects. Based on this bipartite graph, we can extract a production company alliance network in which all coproduction relationships are embedded in this network. Thus, any two production companies are connected by a weighted edge if they have coproduced certain movies. The weight indicates the number of movies in which they have been involved. Similarly, we can construct a distribution company alliance network out of all co-distribution information.

Compared to other industries, the movie industry is ideal to study project alliances from a network perspective with several advantages. First, the boundaries of movie projects in terms of clear lifespan and homogeneous participants are well defined [14]. Alliances in other industries often involve more complicated partnerships along long value chains or supply chains. Second, the mission of a movie project is well focused, and its stages are distinguishable. The financial performance of box office revenue can provide direct measurement for movie projects. However, alliances in other industries are usually multifaceted with tangled structures. Finally, the movie industry possesses unique advantages in openness and data availability, allowing large-scale quantitative analysis and alliance network modelings.

## 3. Hypothesis

In this study, we investigated the following research questions about the performances of movie alliance networks. First, the degree indicates the capabilities of resource pooling in capital and talents, knowledge pooling, as well as reputation built through historical cooperations. Usually, a larger degree is a symbol of great rallying influence in an alliance network. Accordingly, we suggest the first hypothesis:

**Hypothesis** **1.** *Project alliances in which participants have greater degrees in the alliance network positively increase the performance of project alliances.*

For centralities, we consider eigenvector centrality and betweenness centrality in this study. Eigenvector centrality is a local property to describe the overall influence of alliance companies for a given focal company. A higher level of eigenvector centrality indicates that the company is connected to influential companies, but it is itself not necessarily important. In such a case, the company is in a disadvantaged position in which its alliance partners might benefit more from cooperations. This leads to the second hypothesis:

**Hypothesis** **2.** *Project alliances in which participants have higher levels of eigenvector centrality do not positively increase the performance of project alliances.*

Unlike eigenvector centrality, which is a local property, betweenness centrality is a global property to describe the global importance of a company. By definition, betweenness centrality counts the role of a company in the information traffic moving between pairs of companies in the alliance network. However, the betweenness approach requires that paths are the shortest. In an alliance network, for a company, betweenness centrality is largely determined by other, shorter paths for the rest of the alliance network, which is beyond a specific project alliance. Considering this, we tested the third hypothesis:

**Hypothesis** **3.** *Betweenness centralities of participants have no significant influence on the performance of project alliances.*

The structural hole for a given company measures the positional advantages in the alliance network. A company with a structural hole is considered to play as bridges for the less connected parties and thus enjoys complementary information and knowledge of different parts of the networks. In other words, a given company is essential for the alliance network and has better source pooling capability. To test this, we propose the fourth hypothesis:

**Hypothesis** **4.** *Project alliances in which participants have higher levels of structural holes positively increase the performance of project alliances.*

Lastly, for movie alliances of both production and distribution, it is interesting to investigate whether these alliance properties from both alliance networks have the same impact on alliance performance. Therefore, we ask the following research question:

**Research Question** **1.** *Which movie alliance network has an effect on the box office, the production alliance or distribution alliance?*

## 4. Data And Statistics

### 4.1. Data

To conduct an empirical study, we collected our data using several professional movie sources, including MTime.com, a leading professional online movie database and service provider, Boxofficemojo.com, a box office reporting service, and the famous Internet Movie Database (IMDB.com), the most comprehensive online movie database. First, we collected information for 125,627 movies and 86,503 companies from MTime.com. For each movie, we collected all kinds of movie profile information, including genre, budget, cast, awards, nominations, release date, and sequels. To simplify the data, we used the sum of awards and nominations as the award performance. For each company, we also collected a list of all movies it produced and a list of all movies it distributed. It might be a production company or a distribution company. However, it is common for a company to act in both two roles at the same time. In this case, a company is considered both on the production company alliance network and the distribution company alliance network, respectively. Among all 86,503 companies, there were 71,156 production companies that had produced at least one movie and 24,385 distribution companies that had distributed at least one movie. At the intersection, there were 9038 companies that had both the role of production and distribution. We used all this information to generate a production alliance network $N_{P}$ and a distribution alliance network $N_{D}$. In the production alliance network, there were 71,156 production companies and 243,687 coproduction relationships in the same movie projects that were extracted. In the distribution alliance network, there were 24,385 distribution companies and 586,541 co-distribution relationships in the same movie projects that were extracted.

Since movies span over several decades and much information is missing, we further narrowed the movie set by only considering movies released after 2000. By requiring MTime budget information, global box office data on Boxofficemojo, and IMDB review scores, we finally obtained a subset consisting of 1433 movies for further study.

**Figure 1:**
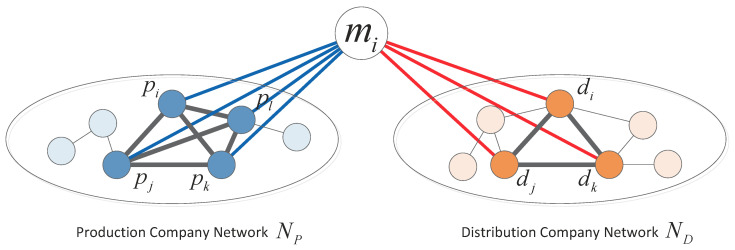
Production company alliance and distribution company alliance for movie $m_{i}$. For this case, in production company network $N_{P}$, four production companies $p_{i}$, $p_{j}$, $p_{k}$, and $p_{l}$ form a production company alliance for $m_{i}$, while in distribution company network $N_{D}$, three distribution companies $d_{i}$, $d_{j}$, and $d_{k}$ form the distribution company alliance for $m_{i}$.

As illustrated in Figure 1, for a movie $m_{i}$, we denoted its production company alliance as $P_{i}$ and distribution company alliance $D_{i}$, respectively. Production company $p_{k}\in P_{i}$, if $p_{k}$ is one of the production companies of movie $m_{i}$. Similarly, distribution company $d_{k}\in D_{i}$, if $d_{k}$ is one of the production companies of movie $m_{i}$. Obviously, $P_{i}$ is part of the production alliance network $N_{P}$, i.e., $P_{i}\subset N_{P}$. Similarly, we also have $D_{i}\subset N_{D}$.

We further calculated the topological properties of production company alliance $P_{i}$ and distribution company alliance $D_{i}$ for movie $m_{i}$ on production alliance network $N_{P}$ and distribution alliance network $N_{D}$, respectively. In our study, the properties of degree, centrality, and structural holes were calculated.

### 4.2. Statistics

Table 1 provides a summary of basic data descriptive statistics. As it shows, we initially collected 125,627 movies, 71,156 production companies, and 24,385 distribution companies. The numbers of alliances in the whole production network and the distribution network were 243,687 and 586,541, respectively. On average, for each production company and distribution company, there were three and 24 alliances, respectively. We saw that both networks were massive and significantly larger than the networks considered in previous studies. After filtering, a set of 903 movies was finally used as sample movies for the regression models.

## 5. Variables And Model

### 5.1. Box Office

Total revenue for a movie includes box office sales and sales generated through a variety of channels like TV, DVDs, and online websites. For theatrical movies, the box office remains the most important indicator for the success of a movie. Most studies of the movie economy adopt the box office to evaluate the financial performance of a movie [15,16,21,23,29,51]. Considering that we focus on the impact of the production and distribution stages, in the same approach of this line of the existing literature, we used the log of box office revenue BO as our dependent variable. All box offices were converted into U.S. dollars.

### 5.2. Movie and Network Variables

We considered two variables. First, in line with many studies on movie performance [52], the movie profile variables included in this study are the movie making budget, the release year, the sequel, and the number of awards. Second, to focus our research on network-related hypotheses, we included network variables of both production and distribution company networks, such as degrees, degree centralities, eigenvalue centralities, betweenness centralities, and structural holes.

#### 5.2.1. Budget

Currently, a movie is an investment-heavy project. The budget of a movie indicating resource availability is a key factor for a movie’s financial success [53]. A big budget movie project can be a good sign of a high quality movie, but might also lead to market failure. Some production companies proactively outline the budget information of a movie as part of the promotion strategy to attract media and public attention. However, it is also common in the movie industry that budget information is not accurately announced for public. For the latter case, estimated budget information provided in movie databases was used [18]. For budget variable Budget, we used the log value.

#### 5.2.2. Sequels

A sequel of a previously successful movie does not guarantee success. However, studies found that sequels perform better than movies on average, with relatively fewer risks [13]. Investigations on cofinancing alliances also found that sequels are less likely to be cofinanced by studios [14]. Since sequels are less risky, they prefer to cofinance movie projects with higher risks [14]. In our sample dataset of 903 movies, there were 142 identified sequels, checked using IMDB information. In our analysis, dummy variable Sequel was introduced and set as one for a sequel movie, otherwise zero.

#### 5.2.3. Awards

The number of award wins from professional societies is an indicator of movie quality, but also a measurement of the human capital for the creative team [6,26]. Usually, wins of leading awards indicate the high quality of a movie. In our analysis, we used the Awards variable as the number of wins. In this study, the Academy Award and other major prestigious awards were included. In total, 1360 wins were identified in 286 movies from 903 sample movies.

#### 5.2.4. Degree

In an alliance network, a company establishes one or multiple alliances with directly-connected partners. The degree of a company, denoted as *k*, thus equals its number of alliances. For a company, a higher degree indicates higher social capital and larger importance in the alliance network. For a movie $m_{i}$, we can calculate the total degrees of all production companies and all distribution companies. For simplicity, we denoted them as PDegree for production alliances and DDegree for distribution alliances, respectively.

#### 5.2.5. Eigenvector Centrality

Eigenvector centrality indicates how a company is connected to other companies with high eigenvector centralities. Technically, the value for a given company is the sum of all connected partners in its alliances. For a movie $m_{i}$, we can calculate the total eigenvector centralities of all production companies and all distribution companies and denote them as PECent and DECent, respectively.

#### 5.2.6. Betweenness Centrality

From another perspective, we calculated betweenness centralities for all production companies and distribution companies. By definition, betweenness centrality describes the global influence of a given node in a network. We denote the two centralities as PBCent and DBCent for production companies and distribution companies, respectively.

#### 5.2.7. Structural Hole

A structural hole indicates how a company is embedded in an alliance network [54]. A company with a higher structural hole value usually holds an important position and enjoys complementary information [55]. Similarly, we calculated the values of structural holes and denote the two centralities as PSH and DSH for production companies and distribution companies, respectively.

### 5.3. Model

In econometrics, linear regression models are widely used to study the impact of independent variables on the dependent variables of interest. Linear regression models are simple and sufficient to quantify the impact in terms of statistical significance and direction. Meanwhile, to be in line with previous empirical studies on the box office that also use linear-regression models, we also modeled our study in a linear regression model. To investigate the influence of the properties of both production and distribution alliance networks on the box office, we used the following regression model to test the aforementioned hypotheses and research question:

$$\begin{array}{c} \log\left(BO\right)=\alpha +\beta_{1}\log\left(Budget\right)+\beta_{2}Sequel+\beta_{3}Award+\beta_{4}PDegree+\beta_{5}CDegree+\beta_{6}PECent+\\ \beta_{7}DECent+\beta_{8}PBCent+\beta_{9}DBCent+\beta_{10}PSH+\beta_{11}DSH+\epsilon . \end{array}$$

## 6. Results And Discussion

In the following, we report the empirical results of the regression model based on the previously-mentioned variables. All network properties were obtained using the NetworkX Python package. Regression analysis was carried out using SPSS with the Linear Regression Module.

Table 2 provides a descriptive statistics of all variables. In this table, it is worth mentioning that the maximum and means of PDegree and DDegree were large with dramatically standard deviations. By looking into the two alliance networks, we found that those movies had large companies as their production companies and distribution companies. These companies are involved in a large number of movie projects in a long history of bringing huge existing alliances, therefore resulting in large alliance degrees. The correlations of all independent variables in the regression model are presented in Table 3. As shown, most correlations were statistically significant at levels of p<0.01 with only a few not significant. We found that Budget, Sequel, and Award were positively correlated with the box office, which agrees with the findings reported in previous studies [15,16,20,36]. The correlations among independent variables indicate certain inter-influences. This leaves potential rooms for dimension reductions. Considering that the objective of this study is to understand how the independent variables contribute to the box office and no variables are excluded for collinearities, so we were able to include all the variables at the same time.

Regression results are presented in Table 4. All results passed statistical tests. The results showed that, except for DDegree, DBCent, and PSH, which were not statistically significant at the level of p<0.1, the other variables were statistically significant at different levels. Among movie profile variables, Budget and Sequel were significant at the level of p<0.01, while Award was significant, but at a relatively lower level of p<0.1. The coefficients were all positive. This agrees with previous studies that movies with larger budgets and sequels are more likely to be successful. However, achievements in art with more award wins slightly contributed to the box office for variables of alliance networks. First, we found that PDegree was positive with a significant level of p<0.01, indicating that movies produced by production companies with greater degrees in production alliance networks are likely to do well at the box office. Hence, Hypothesis 1 is supported. However, DDegree was not significant, so for the case of movie alliances, only PDegree was significant. In other words, degrees of production companies in a production alliance network are important for the box office. Second, PECent was found to be negative at the significant level of p<0.01, while DECent was also negative at a level of p<0.1. The results support Hypothesis 2, which means alliances with higher levels of eigenvector centrality in an alliance network do not positively contribute to the performance of the alliances. For variables of betweenness, results showed that PBCent had a rather small coefficient at a slightly significant level: p=0.074. Moreover, DBCent was not statistically significant. Hence, Hypothesis 3 was supported, that is, that betweenness centralities in alliance networks do not significantly contribute to the performance of the alliances. For the variable of structural holes, we found that PSH was not significant, while DSH was positively correlated at the level of p<0.01 being statistically significant. Therefore, Hypothesis 4 was supported that is that higher levels of structural holes positively increase the performance of the project alliances. For the case of a movie, structural holes in a distribution company network positively contribute to the box office. However, the influence of structural holes in a production company alliance network was not significant.

Finally, we investigated Research Question 1 by comparing the alliance network variables of a production alliance and distribution alliance. We found that a production alliance network had two variables, PDegree and PECent, at a significant level of p<0.01 with larger coefficients, while a distribution alliance network had only one variable, DSH, at the same level with a smaller coefficient. In total, production alliance had three significant variables compared to two significant variables for distribution alliance. We might answer Research Question 1, that both alliance networks had influences on the box office. However, a production company alliance network had relatively more influence than the distribution alliance network.

## 7. Conclusions

Alliances are important networks of cooperations formed by relevant stakeholders. Specifically, project alliances are unique, with limited lifespans, clearly-defined boundaries, multiple stages, and project-oriented missions. Thanks to the public availability of movie project data, the movie industry provides unique opportunities to investigate project alliances. A movie project can be divided into production and distribution stages. Production companies co-finance and organize the making of a movie project before the finished movie is released through market channels by distribution companies. The financial performance of a movie project can be measured by its box office. For a successful movie, all stakeholders involved in the movie project alliance can enjoy the revenue. The box office was found to rely on several determinants of the movie itself [18]. From the perspective of social network theory, studies explored the influence of social network effects at the individual level [26] or production network [46]. The distribution strategy has been explored, but distribution company networks have been less studied [48,56,57].

To investigate the influence of production and distribution alliance networks on the box office, in this work, we used a subset including 903 movies, 2430 production companies, and 2536 distribution companies from the whole dataset. Production company networks and distribution company networks are constructed based on collaborations in the same movie projects. In our final dataset, 903 movies satisfying the data criteria were selected for analysis covering major studios and types up until 2017. Network properties such as degrees, centralities, and structural holes of both alliance networks were calculated for each movie. Taking the box office as the dependent variable and network properties together with movie basic information as independent variables, we built regression models to see how alliance networks influence the box office.

Our results showed that the financial performance of movie alliance projects was significantly influenced by the alliance network properties. Degrees of production companies in a production alliance network were found to positively contribute to the box office, while the degrees of distribution companies in the distribution alliance network had no significant influence on the box office. The eigenvector centralities of both the production and distribution alliance networks did not positively contribute to the box office. However, the betweenness centralities of the two networks did not significantly contribute to the box office. The structural holes of a distribution alliance network significantly and positively contributed to the box office, while the structural holes of a production alliance network did not. From the empirical results, we found that the network properties of two alliance networks had different contributions to the box office. In general, a production alliance network had more influence on the box office than a distribution alliance network.

By using a massive dataset of movie project alliance networks, this study empirically investigated the influence of alliance network topological properties on the performance of project alliances. This work contributes to the literature on box office and alliance networks by considering company-level alliance networks of both production and distribution alliances. The results shed light on how alliance network properties contribute to the financial performance of project alliances. For the case of movies, the two networks contributed to the box office differently. This work adds new evidence to existing studies that mainly focused on individual level cooperation networks or just one of the two alliance networks.

A quantitative understanding of the determinants of financial performance and the influence of both alliance networks is important for movie professionals. Our results have several practical implications for movie industry professionals to achieve better financial performance, i.e., in the box office. First, as usual, a larger budget, better performance in art, i.e., winning awards, and making sequels were more likely to achieve a better box office. Second, choosing alliance networks did matter for the performance of movie project alliances. More specifically, production degrees were important for the box office, but distribution degrees were not. In other words, it is wise to choose production companies with larger degrees in the movie making stage. More resources can be pooled to ensure final box office success. Choosing distribution companies solely with large degrees in distribution company alliance networks had no significant contribution to the box office. Since different companies have various centralities, the present results suggest that it is better to avoid companies with larger eigenvector centralities. Both production and distribution companies did not especially positively contribute to the box office. Larger eigenvector centrality only implied the company had alliances with other companies with even larger centralities. Disadvantaged positions among cooperations hampered the relative bargaining power of the company, offset benefits, and eventually led to negative performance. Unlike the local properties of degrees and eigenvector centralities, global betweenness centralities did not significantly influence the final box office. Therefore, practitioners should focus on local alliances. The results also revealed that the structural holes of distribution company alliance networks instead of production company alliance networks had a significant influence on the box office. This implies that distribution companies with greater structural holes that connect different distribution channels were crucial for the box office.

However, the present work has some limitations that need attention and future exploration. First, the movies considered were only those released after 1964. If we considered a wider period, more movie alliances in different periods could be investigated. It would also be interesting to see how the topological properties and patterns of movie alliances evolve. Second, like the existing literature on the box office that focuses on either individual- or company-level networks, this work studied production- and distribution firm-level alliances. It would be worth trying to consider both levels of alliance networks together to see how the two networks influence each other and how they influence the final box office. Lastly, further work is worthwhile to investigate the topologies of both alliance networks to see how local and global structures behind the properties discussed in the present work influence performance. Meanwhile, in the present work, we included and focused on the independent variables of both production and distribution alliance networks, as well as the topological properties covering degree, eigenvector centrality, betweenness centrality, and structural holes to reflect different aspects of the networks. However, it is worth further investigating other possible models for different combinations of independent variables. Possible dimension reduction approaches could be applied to further simplify the model.

In conclusion, we provided new evidence on the financial performance of production and distribution movie project alliances. With this work, we hope to inspire and stimulate future studies on the performance of alliance networks, as well as the economics of the motion picture industry. 

## Figures and Tables

**Table 1:** Data description. The total dataset was used to construct alliance networks, while the sample dataset was used for regression analyses.

	Total	Sample
Movies	125,627	903
Production companies	71,156	2430
Distribution companies	24,385	2536
Intersection of companies	9038	319
Union of companies	86,503	4647

**Table 2:** Descriptive variable statistics.

Variables	Description	Minimum	Maximum	Mean	Std. Dev.
BO	Box office log	2.640	8.710	6.705	0.854
Budget	Budget log	5.110	9.300	7.518	0.632
Sequel	Sequel dummy	0.000	1.000	0.157	0.364
Award	Number of award wins	0.000	33.000	1.506	3.672
PDegree	Total degrees in production network	1.000	12,500.000	846.461	1283.151
DDegree	Total degrees in distribution network	2.000	44,426.000	14,875.970	10,067.480
PECent	Total eigenvalue centralities in production network	0.000	2.150	0.056	0.225
DECent	Total eigenvalue centralities in distribution network	0.000	3.459	0.723	0.741
PBCent	Total betweenness centralities in production network	0.000	0.095	0.009	0.012
DBCent	Total betweenness centralities in distribution network	0.000	0.093	0.026	0.018
PSH	Total structural holes in production network	0.013	3.704	0.651	0.497
DSH	Total structural holes in distribution network	0.007	2.665	0.481	0.323

**Table 3:** Correlation coefficients.

Variables	BO	Budget	Sequel	Award	PDegree	DDegree	PECent	DECent	PBCent	DBCent	PSH	DSH
BO	1											
Budget	0.517 ***	1										
Sequel	0.3 ***	0.308 ***	1									
Award	0.113 ***	0.09 ***	-0.002	1								
PDegree	-0.014	0.067 **	-0.016	0.013	1							
DDegree	0.282 ***	0.609 ***	0.199 ***	0.204 ***	0.129 ***	1						
PECent	-0.132 ***	-0.025	-0.043 *	-0.045 *	0.925 ***	0.057 **	1					
DECent	0.295 ***	0.595 ***	0.236 ***	0.144 ***	0.07 **	0.74 ***	-0.038	1				
PBCent	0.066 **	0.159 ***	0.031	0.073 **	0.909 ***	0.201 ***	0.75 ***	0.202 ***	1			
DBCent	0.187 ***	0.429 ***	0.114 ***	0.175 ***	0.15 ***	0.884 ***	0.08 ***	0.639 ***	0.199 ***	1		
PSH	-0.09 ***	-0.219 ***	-0.054 *	-0.045 *	-0.026	-0.199 ***	0.001	-0.192 ***	-0.092 ***	-0.139 ***	1	
DSH	0.3 ***	0.381 ***	0.215 ***	0.135 ***	-0.08 ***	0.332 ***	-0.126 ***	0.439 ***	0.004	0.179 ***	0.017	1

* p<0.1; ** p<0.05; *** p<0.01.

**Table 4:** Box office regression results.

Variables	Coeff.	Std. Err.
Budget	0.462 ***	0.052
Sequel	0.152 ***	0.067
Award	0.050 *	0.007
PDegree	0.684 ***	0.000
DDegree	-0.067	0.000
PECent	-0.622 ***	0.335
DECent	-0.087 *	0.052
PBCent	-0.147 *	6.046
DBCent	0.033	3.029
PSH	-0.001	0.049
DSH	0.116 ***	0.085

* p<0.1; ** p<0.05; *** p<0.01.
